# Extraction and Characterization of Microcrystalline Cellulose from *Lagenaria siceraria* Fruit Pedicles

**DOI:** 10.3390/polym14091867

**Published:** 2022-05-02

**Authors:** Muhammad Asif, Dildar Ahmed, Naveed Ahmad, Muhammad Tariq Qamar, Nabil K. Alruwaili, Syed Nasir Abbas Bukhari

**Affiliations:** 1 Department of Chemistry, Forman Christian College (A Chartered University), Lahore 54600, Pakistan; masiftufail143@gmail.com (M.A.); dildarahmed@fccollege.edu.pk (D.A.); tariqqamar@fccollege.edu.pk (M.T.Q.); 2 Department of Pharmaceutics, College of Pharmacy, Jouf University, Sakaka 72388, Aljouf, Saudi Arabia; nkalruwaili@ju.edu.sa; 3 Department of Pharmaceutical Chemistry, College of Pharmacy, Jouf University, Sakaka 72388, Aljouf, Saudi Arabia; sbukhari@ju.edu.sa

**Keywords:** microcrystalline cellulose, *Lagenaria siceraria* pedicles, renewable resources, biowaste utilization

## Abstract

Microcrystalline cellulose (MCC) is a versatile polymer commonly employed in food, chemical, and biomedical formulations. *Lagenaria siceraria* (bottle gourd) fruit is consumed in many parts of the world, and its pedicle is discarded as waste. In the quest for a novel renewable source of the MCC, the present study investigates the extraction and characterization of MCC from the pedicle of *Lagenaria siceraria* fruits. The MCC was extracted by sequentially treating pedicles with water, alkali, bleaching (sodium chlorite), and dilute sulfuric acid (acid hydrolysis). The removal of associated impurities from pedicle fibers was confirmed by Fourier transform infrared analyses. The extracted MCC exhibited a characteristic crystalline structure of cellulose in X-ray diffraction with a 64.53% crystallinity index. The scanning electron microscopy (SEM) showed the variation in the morphology of the fibers and the formation of MCC of approximately 100 µm. The thermogravimetric analysis (TGA) indicated higher thermal stability of MCC. MCC production from biowaste (pedicle) holds potential for application as an emulsifier, stabilizer, and thickener in the chemical, pharmaceutical, and food industries.

## 1. Introduction

The growth in the human population and consumption of plant resources deplete the supply of plant-based materials and result in unpredictable environmental changes [1,2]. The incomplete utilization of the parts of plants leads to the wastage of many valuable natural materials, further aggravating the situation. Therefore, it is the need of the hour to maximize the utilization of the plant parts (fruit, seed, roots, stem, leaves, etc.) for human benefit and the protection of the environment. The fruit of *Lagenaria siceraria* (bottle gourd) is consumed as a vegetable worldwide [3]. According to the Food and Agriculture Organization (FAO) statistics, in 2020, bottle ground was harvested on 2,019,564 hectares worldwide with a total production of 27,962,742 tons [4]. The extracts of the different parts of *Lagenaria siceraria* have been reported to exhibit numerous pharmacological actives, including antimicrobial, antidiabetic, and antioxidant activities [5,6,7]. The pedicle of *Lagenaria siceraria* fruit appears to be composed of strong fibrous material, but this is usually disposed of as waste [8]. Therefore, there is a need to explore the *Lagenaria siceraria* fruit pedicle for the extraction of valuable renewable biopolymers in order to make maximum utilization of the plant resources. 

Cellulose, the most abundant polymer present on this planet, represents an important class of renewable biopolymers [9,10]. Cellulose is commonly employed for versatile industrial applications, attributable to its excellent biodegradability and sustainability [11]. Structurally, cellulose is a linear polymer of the β-d-glucopyranose units linked by the β (1–4) glycosidic bond [12]. Hydrogen bonding and hydrophobic effects are involved in the arrangement of cellulose chains. Hydrogen bonding is a major contributor for defining the crystalline structure and mechanical properties (elastic modulus) of cellulose [13,14]. Although cellulose is insoluble in water, microbes can decompose it [15]. The naturally occurring cellulose microfibrils are arranged into amorphous and crystalline regions [9,10]. The more neat and compact nature of the crystalline domain of cellulose makes it more attractive for industrial applications [16].

Microcrystalline cellulose (MCC) is a fine commercial form of cellulose that is white in color and odorless [17]. Owing to mechanical strength, biodegradability, biocompatibility, large surface area, and compressibility, MCC is used in pharmaceuticals, cosmeceuticals, foods, and textile industries [18,19]. It is employed as a diluent in tablets, suspending agent and viscosity modifier in suspensions and emulsifiers, and water adsorbent in creams and pastes [2,10,20]. Wood fiber is the major source of MCC for industrial utilization. However, MCC can also be obtained from other renewable resources, such as rice husk, an empty bunch of palm oil fruit, tea waste, cotton fabric waste, pomelo peel, bacteria, date seeds, olive fiber, Washingtonia fibers, and Conocarpus fiber [1,20,21,22,23,24,25,26,27,28,29,30]. The cellulose and MCC extracted from different plants and their parts exhibit differences in their physical characteristics such as water absorbability, polymerization, porosity, and crystallinity [31,32]. Three methods are mainly used to prepare MCC by hydrolysis of the cellulose, including acid hydrolysis, enzymatic hydrolysis, and microbial hydrolysis [31]. Among these methods, acid hydrolysis is considered the most suitable as it produces MCC with high crystallinity index [31]. 

The fabrication and characterization of the MCC from the *Lagenaria siceraria* fruit pedicle is not reported previously. Since the plant is cultivated on a large scale and its pedicle is considered waste, the successful fabrication of MCC from this abundantly available source could provide valuable crystalline cellulose material at an affordable cost. Therefore, the present study aims to extract MCC from the *Lagenaria siceraria* fruit pedicle using a serial chemical treatment method. The FTIR, XRD, SEM, EDX, and TGA techniques were employed to characterize the extracted cellulose fibers and confirm the MCC’s formation and purity.

## 2. Materials and Methods

### 2.1. Materials

The fresh pieces of *L. siceraria* fruit were collected from an agricultural farm in Fort Abbas, Punjab, Pakistan, and their pedicles were separated. Sodium hydroxide (NaOH), glacial acetic acid (CH_3_COOH), sodium chlorite (NaClO_2_), and sulfuric acid (H_2_SO_4_) were procured from Sigma-Aldrich (St. Louis, MO, USA).

### 2.2. Extraction of Cellulose Fibers from L. siceraria Pedicle

#### 2.2.1. Water Treatment

The *L. siceraria* fruits were washed with water to remove dirt, and pedicles were separated. The pedicles were then soaked (treated) with water to separate fibrous material from biomass by modifying the previously reported method [33]. For this purpose, approximately 500 g of pedicles were soaked in 5 L water for five days in the open air. Water was refreshed frequently throughout this soaking period until biomass was removed entirely and clear fibrous material was obtained. The resulting fibrous material was then repeatedly washed with double distilled-water and evaporated in the air at room temperature (25 ± 2 °C). Finally, obtained fibers were dried at 110–120 °C in a convection oven to constant weight. The dried fibers were wrapped in a filter paper sheet and placed in a desiccator maintained at 25 ± 2 °C. The percent yield of the fibers was calculated using the following equation:

(1) $$\%\;\mathrm{yield}=\;\frac{\mathrm{wt}.\;\mathrm{of}\;\mathrm{fibous}\;\mathrm{mass}}{\mathrm{wt}.\;\mathrm{of}\;\mathrm{fresh}\;\mathrm{pedicle}}\times 100$$

#### 2.2.2. Alkali Treatment

To remove hemicellulose and other alkali-soluble components, the pedicle fibers obtained after water treatment were treated with an alkali [34]. For this purpose, 4 g of fibers were added in a round bottom flask (0.5 L), and 80 mL NaOH (5% *w*/*v*) solution was added. The contents were refluxed for 3 h at 180 °C on a hotplate. Fibers obtained were washed with distilled water and soaked in 0.5 L distilled water at 25 ℃ for 3 h. The pH of the water was neutralized with acetic acid (3%). The fibers were dried in a fume hood at room temperature and cut into small pieces. The alkali treatment was repeated 4 times.

#### 2.2.3. Bleaching

After alkali treatment, pedicle fibers were bleached to remove lignin and decolorize. For this purpose, the acetic acid buffer was used with sodium chlorite. A solution of 2.7 g sodium hydroxide in a few mL of water was mixed with 7.5 mL glacial acetic acid in a 100 mL measuring flask, and the flask was filled with water up to the mark. The pH of the buffer was adjusted to 4.6. The sodium chlorite solution (1.7% *w*/*v*) was prepared in distilled water and mixed with an equal buffer volume. For bleaching, the reflux method was used [34]. Approximately 3 g of alkali-treated fibers were added in a 250 mL flat-bottom flask with 120 mL buffer and sodium chlorite mix. The reflux was carried out for 2.5 h at 120–130 °C with vigorous stirring. After every 30 min, the solution was diluted with 10 mL of distilled water to reduce the temperature. The opening was open for the evacuation of chlorine gas with colored substances. After bleaching, the mix was cooled to ambient temperature (around 25 ℃) and filtered. The residue was dried at 120 °C to evaporate water vapors and impurities. The bleaching process was repeated 5 times to obtain completely decolorized fibers.

### 2.3. Fabrication of MCC

Cellulose microcrystals were fabricated by the acid hydrolysis of the fibers obtained by bleaching [35]. For this purpose, approximately 4 g of bleached fibers were added to a flat-bottom (250 mL) flask containing distilled water (10 mL), refluxed with 160 mL of 40% H_2_SO_4_, and left at about 85–95 °C for 40–50 min. When thick milky white clouds were formed in the flask, a small amount of cold deionized water was added, and heating was stopped. The flask was shifted to an ice bath, and after every 2 min, 100 mL of deionized water was added until the reaction stopped completely. After that, the sample was gravity-filtered through Whatman filter paper 41 and thoroughly washed with ice-cold deionized water to ensure the complete removal of hydrolysis material. The samples were centrifuged at 400 rad/s for 15 min, and sonicated at the amplitude power of 40% for 8–10 min. Finally, the samples were freeze-dried (BK-FD-12P, Biobase Co., Ltd., Jinan, Shandong, China) to obtain microcrystalline cellulose (MCC). 

### 2.4. Characterization

#### 2.4.1. Fourier Transform Infrared Spectroscopy

ATR-FTIR analyses were performed using an FTIR spectrophotometer (Carry 630, Agilent Technologies, Santa Clara, CA, USA) equipped with a diamond attenuated total reflectance (ATR) assembly. The pedicle fibers (water-treated, alkali-treated, bleached) and MCC were directly placed on the ATR sampler, and FTIR spectra were acquired in the 4000 to 650 cm^−1^ wavenumber range.

#### 2.4.2. X-ray Diffraction (XRD) Analysis

The alkali-treated pedicle fibers and MCC were analyzed by Powder XRD using X-ray diffractometer (PANalytical X’Pert PRO, Malvern Panalytical, Malvern, UK) with Cu Kα (λ = 0.15418 nm) incident radiation in the 2*θ* range 10–80°. The crystallinity index *(CrI*) of the MCC was determined according to Equation (2) [28]:

(2) $$CrI\left(\%\right)=\frac{I_{\left(0\;0\;2\right)\;}-I_{am}}{I_{\left(0\;0\;2\right)}}\times 100$$

where *I*_(0 0 2)_ is the maximum intensity of the crystalline pattern of cellulose located at 2*θ* = 22.7°, and *I_am_* represents the intensity of the amorphous domain at 2*θ* = 18.5°.

#### 2.4.3. Scanning Electron Microscopy and Energy-Dispersion X-ray Analyses 

The microstructures and surface morphologies of the carbon-coated pedicle fibers (alkali-treated and bleached) and MCC were observed by variable pressure SEM (Vega 3 LMU, TESCAN, Brno, Czech Republic). Micrographs were taken at different magnifications. EDX of samples was recorded along with SEM analysis to detect elements present in cellulose samples after alkali treatment, bleaching, and acid hydrolysis. For this purpose, an EDX detector (Oxford Instruments, Abingdon, UK) coupled with SEM was used.

#### 2.4.4. Thermal Analysis 

The thermal degradations of pedicle fibers (alkali-treated and bleached, and MCC samples were studied using a thermogravimetric analyzer (TGA-50, Shimadzu, Kyoto, Japan). For this purpose, ~5 mg of sample were sealed in the standard pans and subjected to heat from ~30 to 500 °C, at 15 °C/min heating rate with a constant flow of nitrogen gas (20 mL/min). The thermogravimetric (TG) curves were presented as percentage loss in mass versus temperature (°C). The derivatives of TG curves (DTG) were calculated using the inbuilt instrument software.

## 3. Results and Discussion

### 3.1. Extraction of MCC from Lagenaria siceraria Pedicle

**Figure 1:**
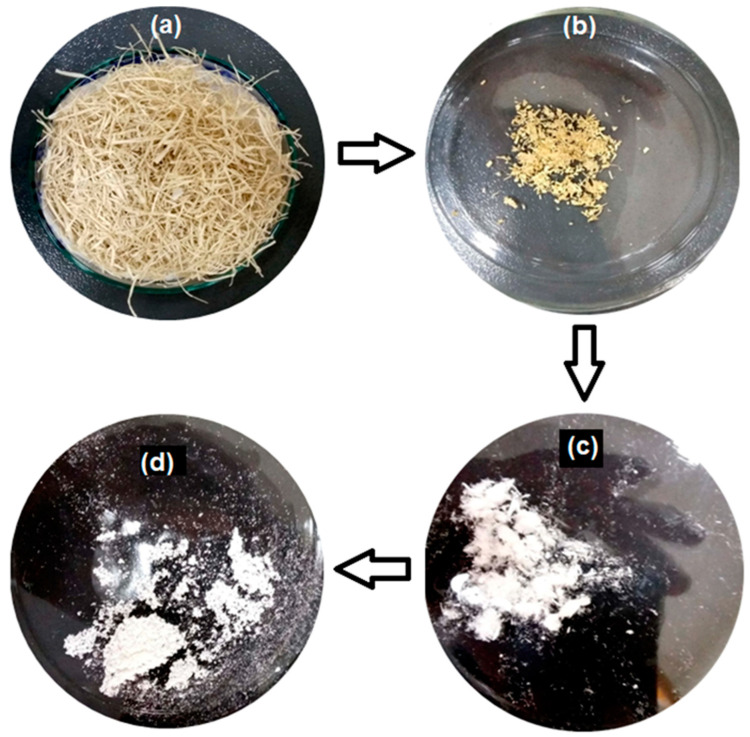
Optical images of *Lagenaria siceraria* fruit pedicle fibers after (**a**) water treatment, (**b**) alkali treatment, (**c**) bleaching, and (**d**) acid hydrolysis (MCC).

The optical images captured at different stages during the extraction of MCC from *Lagenaria siceraria* fruit pedicle are depicted in Figure 1. In the first step of extracting cellulose, fruit pedicles were treated with water to eliminate biomass. The soaking of the pedicles in water allowed microorganisms to act on the pedicle and remove the non-cellulosic mass from the fibers [33]. As a result of water treatment, the shining and yellowish fibers were obtained (Figure 1a). Approximately 150 g of the dried fibers were obtained from 500 g of fresh pedicles (~30% yield). The cellulose fibers obtained from the plant material are linked with hemicellulose and lignin, which can be solubilized and hydrolyzed by the alkali solution, unlike cellulose which is insoluble in an alkali [33]. After alkali treatment, the extracted pedicle fibers were yellow (Figure 1b). The decolorizing effect of bleaching is dependent on pH and temperature, best at pH 4.56 and 120–130 °C. Bleaching reduced fiber size and produced microfibrils (Figure 1c). Acid hydrolysis with sulfuric acid is a well-established method for fabricating MCC from cellulose fibers [31,36]. The acid treatment cleaves cellulose fibers into numerous MCC, removing the amorphous region from fibers [22,33,34]. However, the shape and size of crystals depend on selecting appropriate hydrolysis conditions and the source of cellulose (plant part) [22]. Initially, different conditions (concentrations of H_2_SO_4_, temperature, and time) were applied to produce MCC. It was determined that 40% H_2_SO_4_ at 80–90 °C for 50 min was most suitable for processing *L. siceraria* pedicles fibers. The use of a higher concentration of acid resulted in samples burning. These hydrolysis conditions differed from those previously reported for MCC production from different cellulose sources, supporting the argument that hydrolysis conditions are source-specific [32,37,38]. 

### 3.2. Characterization

#### 3.2.1. Fourier Transform Infrared Spectroscopy

**Figure 2:**
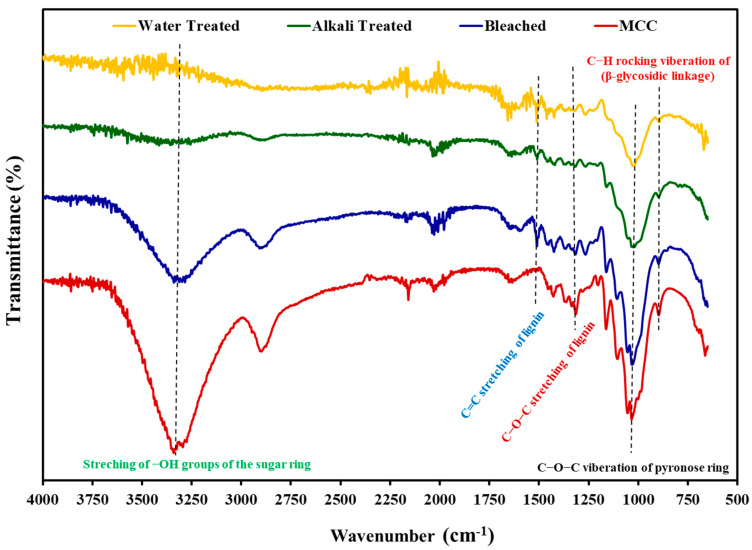
Comparison of FTIR spectra. The absence of designated peaks corresponding to stretching bands of lignin presented in ovals, and the presence of stretching bands of sugar ring (dotted lines) and glycosidic linkage (rectangular box), confirm the formation of lignin-free cellulose microcrystals after acid hydrolysis.

The IR spectra of water-treated, alkali (NaOH)-treated, bleached (pedicle fibers), and acid-hydrolyzed (MCC) *L*. *siceraria* pedicles exhibited the effect of chemical treatment on the structure of the extracted fibers and confirmed the removal of lignin and hemicellulose from the fabricated MCC (Figure 2). In the spectra of the pedicle fibers (water-treated, alkali-treated, and bleached), C=C stretching peak was found at ~1500 cm^−1^ (due to the presence of lignin). The presence of this peak in the bleached sample indicates that lignin was not completely removed from the pedicle fibers [29]. However, this peak was absent in spectra of MCC samples, suggesting that lignin was absent in the MCC due to hydrolysis of the lignin cellulose bonds by the acid treatment [28]. The lignin removal was further confirmed by the absence of the C–O–C (aryl-alkyl ether of lignin) peak around 1250 cm^−1^ in MCC spectra. 

The peaks attributed to the C–H groups stretching, O–H stretching (of absorbed water), and vibration of pyranose ring (C–O–C) and rocking vibrations of C–H (β-glycosidic linkage) were found around 2900, 1650, and 1020−1035, and 892 cm^−1^, respectively, in all tested samples. The peaks from 3400−3200 cm^−1^ can be ascribed to the stretching of the O–H groups of the sugar ring. Moreover, the intensity of the peak increased with sequential chemical treatment, which could be ascribed to the relative increase in the O–H group in samples with treatment and increased exposure of the O–H group with the reduction in size [28,37]. Furthermore, an increase in the sharpness of the C–O–C vibration (1030 cm^−1^) and β-glycosidic linkage (892 cm^−1^) peaks was observed after alkali treatment, bleaching, and acid hydrolysis (MCC), which might be ascribed to the change in the orientation of the cellulose [28,29]. 

The FTIR results indicated that hemicellulose and lignin were detached from fibers extracted from pedicles after alkali, bleach, and acid hydrolysis treatment. The FTIR spectra of acid-hydrolyzed sample (MCC) exhibited characteristic peaks attributed to functional groups of pure cellulose. These FTIR spectra of MCC obtained from *Lagenaria siceraria* pedicles were in close agreement with the previously reported spectra of the MCCs obtained from different sources [20,26].

#### 3.2.2. X-ray Diffraction Analysis

**Figure 3:**
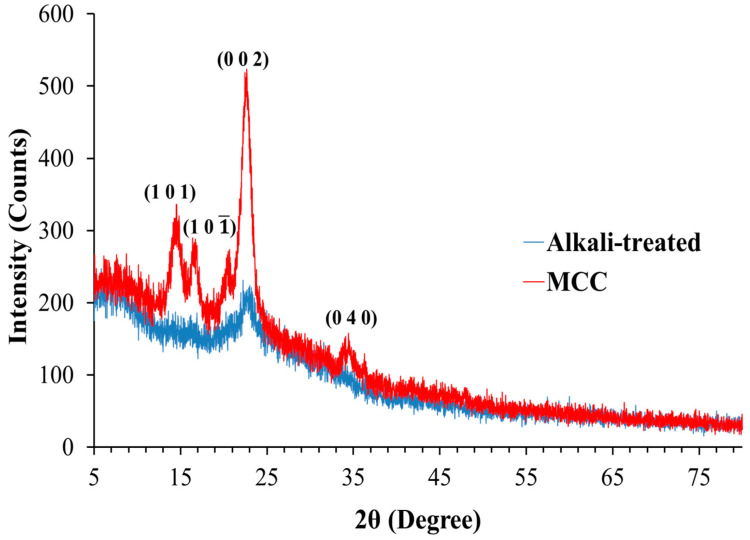
The X-ray diffractograms of alkali-treated fibers and MCC obtained from the *Lagenaria siceraria* fruit pedicle.

Cellulose contains numerous OH groups that interact by forming intra- and intermolecular hydrogen bonds. The hydrogen bonding results in an ordered arrangement of molecules, and cellulose exhibits a crystalline structure. The crystallographic patterns of fibers obtained from the pedicle analyzed after alkali treatment and acid hydrolysis (MCC) are presented in Figure 3. The X-ray diffractogram of alkali-treated fibers exhibited a small peak at 2θ = ~22.9°, indicating that alkali-treated fibers were amorphous in nature. In contrast, X-ray diffractogram of MCC exhibited 2θ = 14.8°, 16.4°, 22.7°, and 34.5°, ascribed to (1 0 1), (1 0 $\bar{1}$), (0 0 2), and (0 4 0) crystallographic planes, respectively [37,39].

The XRD findings indicate that MCC fabricated from pedicle comprised characteristic crystalline structure of cellulose I. [29]. The crystallinity indexes (*CrI*) of the alkali-treated fibers and MCC were 40.43% and 64.53%, respectively. These *CrI* values indicate that the crystallinity of the cellulose (MCC) extracted from the pedicle was increased after acid hydrolysis. Cleavage of the glycosidic linkage due to the hydrolytic action of acid allows cellulose crystals to align, resulting in increased crystallinity index and sharpening of XRD peaks [40]. These results are in close agreement with previous studies, where an increase in the *CrI* was reported after acid hydrolysis [26,40]. The *CrI* of the MCC isolated in this study is within the range of the *CrI* of commercial MCC, which can be 55 to 80% [27]. Previous studies reported different *CrI* for MCC extracted from different plant sources that might be attributed to the difference in nature of the plant fibers and the conditions used for isolation [26]. The *CrI* of the MCC obtained in this study (64.53%) is in close agreement with *CrI* of MCC obtained from oil palm fronds (62.5%) [14] and is higher than *CrI* MCC obtained from *Ensete glaucum* (Roxb.) (53.4%) and pomelo peel (40.53%) [2,26].

#### 3.2.3. Scanning Electron Microscopy and Energy-Dispersion X-ray Analyses

**Figure 4:**
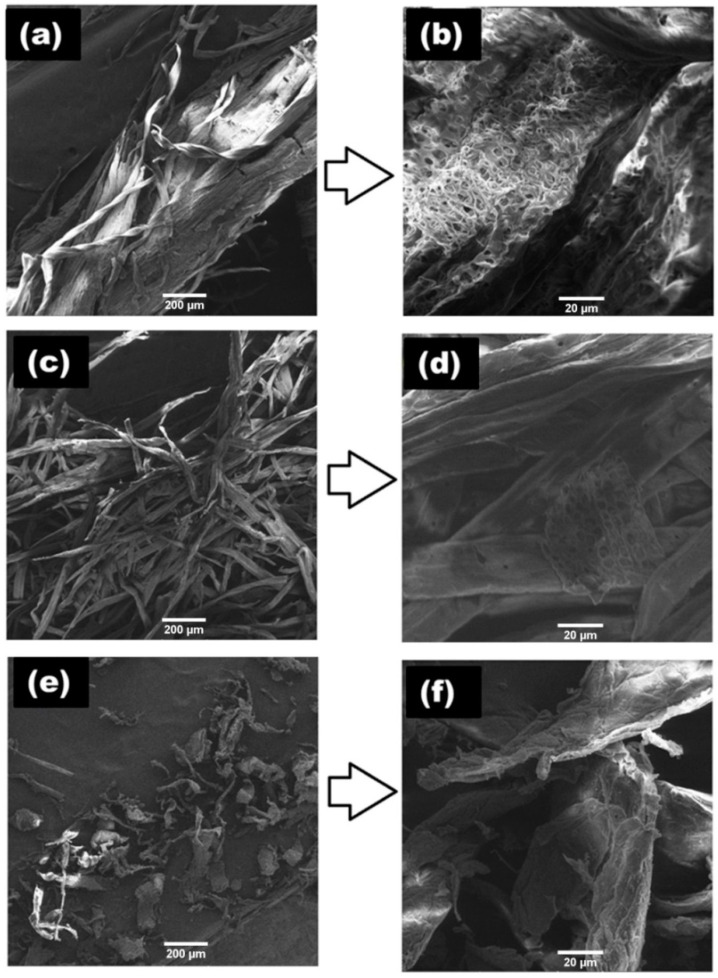
SEM images indicating variation in morphology of alkali-treated (**a**,**b**), bleached (**c**,**d**), and acid-hydrolyzed (**e**,**f**) samples at a resolution of 100× (**a**,**c**,**e**) and 1000× (**b**,**d**,**f**), respectively.

The SEM micrographs of the pedicle fibers (alkali-treated and bleached) and MCC indicated variation in morphology fibers and MCC extracted from *L. siceraria* fruit pedicle (Figure 4). In alkali-treated fibers, a large bundle of fibers was observed at low magnification (Figure 4a), while highly intertwined fibers were observed at higher magnification (Figure 4b). The bundles of fiber indicate the presence of lignin which forms linkages with cellulose and acts as a binder for cellulose fibers [28,36]. After bleaching, large bundles of cellulose fibers were converted into smaller individual fibers (Figure 4c,d). This conversion indicates that bleaching reduced lignin. After acid hydrolysis, cellulose fibers were converted into irregular-shaped microparticles/MCC (Figure 4e,f), which may be due to the strong hydrolysis effect of the acid at high temperature that removed lignin and the amorphous region of cellulose and formed MCC. Thus, the acid hydrolysis of the fibers produces MCC with irregularly shaped particles of ~100 µM size. Similar patterns of morphological changes were observed during the extraction of the MCC cellulose from bamboo fibers and palm oil biomass water [28,41].

The results of EDX analysis of cellulose extracted from the *Lagenaria siceraria* fruit pedicle at different chemical treatment stages are given in Table 1 and Figure S1. The results suggest that carbon (C) and oxygen (O) were major elements present in MCC that are the characteristic components of cellulose [28,36]. MCC exhibited a higher percentage of carbon (~50.4%) and a lower percentage of oxygen (~49.5%) as compared to the other two samples (alkali-treated and bleached fibers). This relative decrease in oxygen content can be suggested as owing to the removal of oxygen-containing non-cellulosic components of the cell wall (lignin, hemicellulose, ferulic acids, and uronic acids) upon bleaching and acid hydrolysis [28]. Moreover, the percentage of impurities was almost negligible in the tested sample.

#### 3.2.4. Thermal Analysis (TGA)

**Figure 5:**
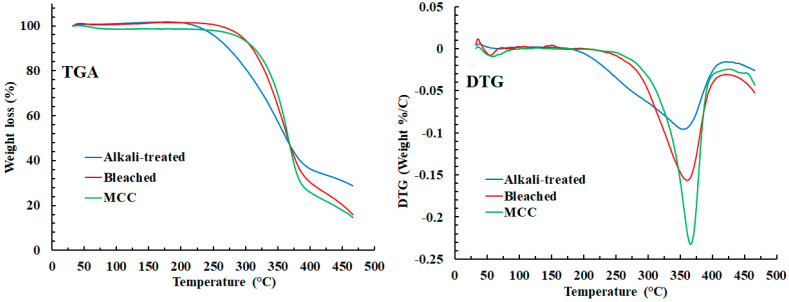
TG and DTG curves of alkali-treated, bleached, and MCC samples, indicating that higher thermal stability (*T_max_*) is exhibited by acid-hydrolyzed MCC than alkali-treated and bleached pedicle fibers.

The results of the TGA of pedicle fibers (alkali-treated and bleached) and MCC are shown in Figure 5. These TGs suggest that all tested samples underwent single-step degradation with weight loss of ~63.2, 67.7, and 70.1% for alkali-treated, bleached, and MCC samples, respectively. The single-step degradation of cellulosic material is likely due to the depolymerization of fiber and decomposition of the carbohydrate backbone [42,43]. The onset of degradation for alkali-treated, bleached, and MCC samples was at 209.5, 236.1, and 245.1 °C, respectively. The higher onset temperature for bleached pedicle fibers and MCC degradation can be attributed to a higher cellulosic content that resists degradation at a lower temperature. The MCC exhibited the highest onset degradation temperature due to its crystalline nature [28]. In DTG curves, the temperatures for maximum thermal degradation (*T_max_*) for alkali-treated, bleached, and MCC samples were 358.7. 359.9, and 366.2 °C, respectively, which suggest higher thermal stability of the cellulose fibers. The DTG curve of MCC was sharpest, indicating higher purity of the cellulose and confirming the removal of impurities after acid hydrolysis [28,35]. These findings of TGA indicate that MCC from *L. siceraria* fruit pedicle has high thermal stability for industrial applications.

## 4. Conclusions

In this study, the MCC successfully extracted the *Lagenaria siceraria* fruit pedicle through sequential alkali treatment, bleaching, and acid hydrolysis. The optimal conditions for acid hydrolysis were 40% H_2_SO_4_ at 80–90 °C for 50 min. The FTIR analysis confirmed the removal hemicellulose and lignin from MCC, after acid hydrolysis of the pedicle fibers. XRD and SEM analyses also suggested MCC fabrication by acid hydrolysis with 64.53% crystallinity index and ~100 µm size. The MCC exhibited the highest thermal stability in TGA. These findings suggest that the *Lagenaria siceraria* fruit pedicle (biowaste) could be a cost-effective and renewable source of the MCC that can be further explored for various industrial applications.

## Figures and Tables

**Table 1:** Elemental composition of alkali-treated, bleached, and MCC samples.

Sample	Weight%		Atomic%	
	Carbon	Oxygen	Carbon	Oxygen
Alkali-treated	45.79	54.21	52.95	47.05
Bleached	46.77	53.23	53.92	46.08
MCC	50.42	49.58	57.53	42.47

## Data Availability

Not applicable.

## Supplementary Materials

The following supporting information can be downloaded at: https://www.mdpi.com/article/10.3390/polym14091867/s1, Figure S1: EDX spectra of alkali-treated bleached and MCC samples obtained from *Lagenaria siceraria* fruit pedicles.
