# Supplementary material for: Extraction and Characterization of Microcrystalline Cellulose from Lagenaria siceraria Fruit Pedicles
Source: Polymers (Basel). 2022 May 2;14(9):1867. doi: 10.3390/polym14091867 (PMC9101574; doi:10.3390/polym14091867)
Supplement: Supplementary file 1 [file polymers-14-01867-s001.zip › polymers-1685413-supplementary.pdf]

Supplementary Material

Extraction and Characterization of Microcrystalline Cellulose from *Lagenaria siceraria* Fruit Pedicles

Muhammad Asif [1], Dildar Ahmed [1], Naveed Ahmad [2,*], Muhammad Tariq Qamar [1], Nabil K. Alruwaili [2] and Syed Nasir Abbas Bukhari [3]

[1] Department of Chemistry, Forman Christian College (A Chartered University), Lahore 54600, Pakistan; masiftufail143@gmail.com (M.A.); dildarahmed@fccollege.edu.pk (D.A.); tariqqamar@fccollege.edu.pk (M.T.Q.)
[2] Department of Pharmaceutics, College of Pharmacy, Jouf University, Sakaka 72388, Aljouf, Saudi Arabia; nkalruwaili@ju.edu.sa
[3] Department of Pharmaceutical Chemistry, College of Pharmacy, Jouf University, Sakaka 72388, Aljouf, Saudi Arabia; sbukhari@ju.edu.sa
* Correspondence: nakahmad@ju.edu.sa

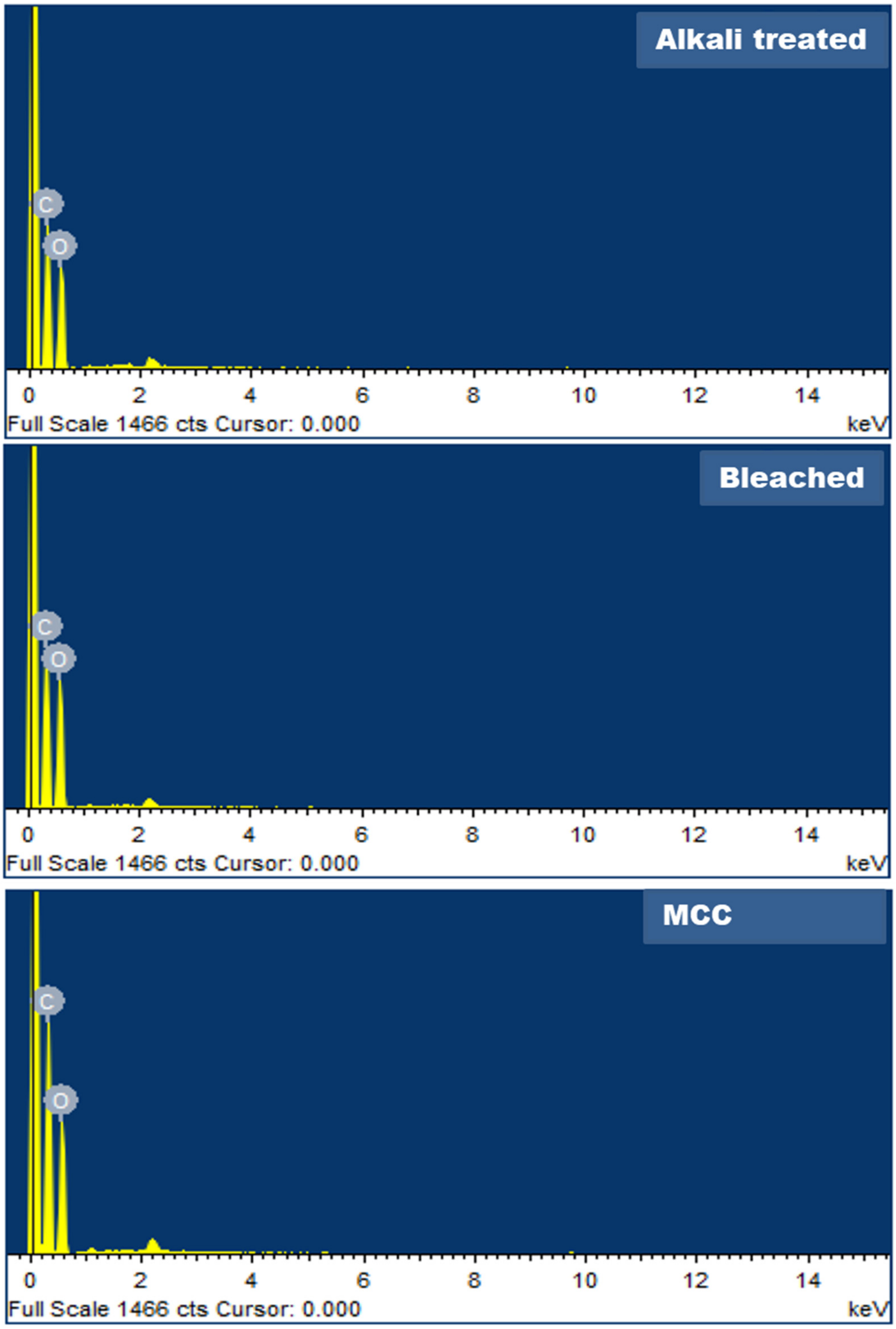

Figure S1: EDX spectra of alkali-treated, bleached and MCC samples obtained from *Lagenaria siceraria* fruit pedicles.
